# Capacity building for evidence-based decision making in local health departments: scaling up an effective training approach

**DOI:** 10.1186/s13012-014-0124-x

**Published:** 2014-09-24

**Authors:** Julie A Jacobs, Kathleen Duggan, Paul Erwin, Carson Smith, Elaine Borawski, Judy Compton, Luann D’Ambrosio, Scott H Frank, Susan Frazier-Kouassi, Peggy A Hannon, Jennifer Leeman, Avia Mainor, Ross C Brownson

**Affiliations:** Prevention Research Center in St. Louis, Brown School, Washington University in St. Louis, St. Louis, MO USA; Department of Public Health, University of Tennessee, Knoxville, TN USA; Case Western Reserve University, Cleveland, OH USA; Michigan Public Health Training Center, University of Michigan, Ann Arbor, MI USA; Northwest Center for Public Health Practice, University of Washington, Seattle, WA USA; Prevention Research Center of Michigan, University of Michigan, Ann Arbor, MI USA; Health Promotion Research Center, University of Washington, Seattle, WA USA; Center for Health Promotion and Disease Prevention, University of North Carolina, Chapel Hill, NC USA; Division of Public Health Sciences, Alvin J. Siteman Cancer Center, Washington University School of Medicine, St. Louis, MO USA

**Keywords:** Evidence-based public health, Public health workforce training

## Abstract

**Background:**

There are few studies describing how to scale up effective capacity-building approaches for public health practitioners. This study tested local-level evidence-based decision making (EBDM) capacity-building efforts in four U.S. states (Michigan, North Carolina, Ohio, and Washington) with a quasi-experimental design.

**Methods:**

Partners within the four states delivered a previously established Evidence-Based Public Health (EBPH) training curriculum to local health department (LHD) staff. They worked with the research team to modify the curriculum with local data and examples while remaining attentive to course fidelity. Pre- and post-assessments of course participants (n = 82) and an external control group (n = 214) measured importance, availability (*i.e.*, how available a skill is when needed, either within the skillset of the respondent or among others in the agency), and gaps in ten EBDM competencies. Simple and multiple linear regression models assessed the differences between pre- and post-assessment scores. Course participants also assessed the impact of the course on their work.

**Results:**

Course participants reported greater increases in the availability, and decreases in the gaps, in EBDM competencies at post-test, relative to the control group. In adjusted models, significant differences (p < 0.05) were found in ‘action planning,’ ‘evaluation design,’ ‘communicating research to policymakers,’ ‘quantifying issues (using descriptive epidemiology),’ and ‘economic evaluation.’ Nearly 45% of participants indicated that EBDM increased within their agency since the training. Course benefits included becoming better leaders and making scientifically informed decisions.

**Conclusions:**

This study demonstrates the potential for improving EBDM capacity among LHD practitioners using a train-the-trainer approach involving diverse partners. This approach allowed for local tailoring of strategies and extended the reach of the EBPH course.

## Background

An evidence-based decision making (EBDM) process in public health involves making use of the best available scientific evidence, engaging communities in assessment and decision making, applying planning frameworks, conducting sound evaluations, and disseminating results through appropriate channels [1,2]. In recent years, efforts have been made to establish more uniform guidelines related to EBDM for public health practitioners and agencies. For example, based on recommendations of the Institute of Medicine, *Core Competencies for Public Health Professionals* emerged to define ‘a set of skills desirable for the broad practice of public health’ [3]. Additionally, the Public Health Accreditation Board (PHAB) is leading a voluntary accreditation effort in the United States to establish national achievement standards for health departments, including such requirements as ‘maintain a competent public health workforce’ (Domain 8) and ‘contribute to and apply the evidence base of public health’ (Domain 10) [4]. Funders are increasingly interested in supporting projects that are evidence-based and may soon prioritize funding accredited health departments to ensure effective use of their funds [5-7].

Based on literature in the emerging field of dissemination and implementation research [8,9], the scale-up of effective workforce capacity-building approaches is a key need for research and practice [10]. The public health workforce is transdisciplinary by nature and represents diverse educational backgrounds and job types [11-14]. There is a need for comprehensive training programs that build and maintain common skillsets and language among public health practitioners to accomplish EBDM goals [15,16]. The Prevention Research Center in St. Louis (PRC-StL) developed an Evidence-Based Public Health (EBPH) training course in 1997 with support from the Centers for Disease Control and Prevention and the World Health Organization. To date, the EBPH course has been offered to over 1,240 participants by faculty associated with the PRC-StL. Course content aligns closely with core competencies of public health [2,3,17] and covers specific skills to improve public health practice [18].

A series of mixed methods evaluations have shown that the EBPH course is effective in improving self-reported measures of knowledge, skill, and ability [16,19,20]. The present study represents the first evaluation of this course curriculum with a quasi-experimental design. A train-the-trainer approach was used to engage partners in four states in efforts to improve EBDM capacity among local health department (LHD) practitioners. Much of the research on improving EBDM has been focused on state-level practitioners, even though gaps in skills are higher at the local level [21,22].

## Methods

### Selection of intervention states

Intervention activities were delivered in four U.S. states: Michigan, North Carolina, Ohio, and Washington. Prevention Research Centers (PRCs) in these states formed partnerships with either Public Health Practice Based Research Networks (PBRNs) or Public Health Training Centers (PHTCs) to conduct capacity-building activities for their state’s local health departments. For a PRC to be eligible for this study, the following criteria had to be met: a PBRN and/or PHTC existed in the same state; the PRC-PBRN or PRC-PHTC pair had a track record of productive collaboration; there were at least 30 LHDs in the state; the PRC had a strong mission and track record in training public health practitioners; and it had not already conducted extensive trainings in EBPH with LHD practitioners. The following PRC-PBRN/PHTC pairs were chosen:

1. University of Michigan PRC of Michigan; Michigan PHTC.
2. University of North Carolina at Chapel Hill Center for Health Promotion and Disease Prevention; Southeast PHTC.
3. Case Western Reserve University PRC for Healthy Neighborhoods; Ohio PBRN.
4. University of Washington Health Promotion Research Center; Northwest Center for Public Health Practice, PHTC.

### Development of intervention activities

The intervention primarily involved the delivery of the EBPH training course. However each PRC-PBRN/PHTC team was also expected to provide at least one additional capacity-building activity with the attendees of the training based on the needs of their course’s participants (*e.g.*, technical assistance with community assessment, grant proposal development, program development, implementation, or evaluation; practicum opportunities for public health/preventive medicine students and LHDs).

The EBPH curriculum consists of nine modules (see next section for a list of modules and learning objectives) and adheres to adult learning principles (*i.e.*, learning through problem solving and active involvement, integrating the experiences of faculty and participants into course discussions) [14,23]. Seven of the nine modules (excluding Modules 1 and 6) include interactive exercises in which participants work in small groups (*e.g.*, using local data to develop a concise problem statement, searching PubMed for literature on a specific topic, developing an action plan based on a logic model).

At least two representatives from each state traveled to St. Louis in November 2012 for a 2.5-day ‘train-the-trainer’ workshop conducted by members of the research team. The workshop included review of the EBPH course curriculum developed by the PRC-StL. In collaboration with previous EBPH trainers, new trainers discussed sources of local data and examples of successful programs and policies to be used to modify the curriculum. Attendees also received detailed information on the administrative process for planning and conducting a successful training (*e.g.*, registration processes, site selection, preparation of course materials). Over the next six months, the research team provided state partners with technical assistance as they modified the curriculum for local relevance while being attentive to course fidelity, ensuring consistency with the original curriculum and with what was delivered in other states.

One course was conducted in each of the four states during the months of April-June 2013, and 130 participants completed the course. North Carolina and Ohio conducted 3.5-day in-person trainings. To reduce travel costs and the burden of time away from the office for attendees, the other states opted to deliver three of the nine modules via interactive webinars (Michigan: Modules 3, 5, 7; Washington: Modules 1, 5, 7) with the remaining modules delivered in two days of in-person sessions. The PRC-PBRN/PHTC partners, with help from their state health departments, recruited participants through website postings, announcements and flyers at conferences, and emails to various public health electronic mailing lists. Each state had a waiting list for their training course.

### EBPH modules and learning objectives

Module 1: Introduction:

1. Understand the basic concepts of evidence-based decision making.
2. Introduce some sources and types of evidence.
3. Describe several applications within public health practice that are based on strong evidence and several that are based on weak evidence.
4. Define some barriers to evidence-based decision making in public health settings.

Module 2: Community assessment:

1. Understand the importance of conducting a community assessment.
2. Understand the types of data that are appropriate for assessing the needs and assets of the population/community of interest.
3. Understand the major steps in the community assessment process.

Module 3: Quantifying the issue:

1. Measure and characterize disease frequency in defined populations using principles of descriptive epidemiology and surveillance.
2. Find and use disease surveillance data presently available on the Internet.

Module 4: Developing a concise statement of the issue:

1. Understand the overall strategic planning process for setting priorities in public health.
2. Understand a criterion for the components of a sound problem statement.
3. Develop a concise written statement of the public health problem, issue or policy under consideration in a measurable manner.

Module 5: Searching and summarizing scientific literature:

1. Understand the process used in systematic reviews and identify a key source (*e.g.*, the Community Guide).
2. Use recommended guidelines for searching the scientific literature.

Module 6: Developing and prioritizing options:

1. Identify methods for prioritizing program and policy options (Types 1, 2, and 3).
2. Explore the role of creativity and group processes in developing intervention options.
3. Understand when and how to adapt interventions for different communities, cultures, and settings.

Module 7: Economic evaluation:

1. Know the differences between types of economic evaluations: cost-benefit, cost-utility, and cost-effectiveness analysis.
2. Understand key terms in economic analysis.
3. Be able to use economic evaluation studies to justify, prioritize, and implement prevention and treatment strategies.

Module 8: Developing an action plan and building a logic model:

1. Identify key characteristics and principles in successful action planning, including the role of coalitions/partnerships.
2. Identify the steps in program planning.
3. Understand the purpose and use of logic models.
4. Describe steps used in constructing logic models.

Module 9: Evaluating the program or policy:

1. Understand the basic components of program evaluation.
2. Understand the various types of evaluation designs useful in program evaluation.
3. Understand the concepts of measurement validity and reliability.
4. Understand the contributions of both qualitative and quantitative data to the evidence based process.
5. Understand some of the methods used in qualitative evaluation.
6. Understand organizational issues in evaluation.

### Selection of control group

Control group selection began with a merged database of two national surveys previously conducted by the research team. In October-December 2012, a random sample of 1,067 U.S. LHDs was drawn from the database of 2,565 LHDs maintained by the National Association of County and City Health Officials, resulting in available pre-test data from 517 LHD directors or their designees (54% response rate) [24]. Respondents of this survey identified program managers within their same LHD, resulting in the collection of 332 additional responses from December 2012 to February 2013 (67% response rate) [25]. The focus of these surveys was to identify evidence-based training, practices, and related decision-making activities.

A subsample of the merged directors’ and program managers’ surveys (n = 849) was selected to be retested to serve as the control group. Because baseline surveys found that governance structure and population of jurisdiction were significantly related to administrative evidence-based practices [24,25], we used these variables, along with job position, to guide our sample selection. Because all LHDs in the four intervention states are locally governed, the sample was first restricted to respondents whose LHD followed a localized (decentralized) governance structure. Next, we eliminated anyone who attended or had a colleague who attended the EBPH training. Finally, we stratified the remaining group by job position and population of jurisdiction and selected participants to best parallel the intervention group’s stratification at a 3:1 ratio. Despite the improved balance across control and intervention groups, they still significantly differed (p < 0.05) on these matching variables, as there were not enough controls to match in the higher population categories and the lower job positions. These differences were therefore controlled for in the analysis. Of those invited to the control group (n = 330), 40% came from the directors’ survey and 60% from the program managers’ survey.

### Questionnaire development and testing

Baseline surveys were identical for control and intervention groups; development of this instrument is described previously [24,25]. From this baseline instrument, the post-test questionnaire retested a set of questions related to perceived importance and availability of EBDM competencies. This set of questions was originally informed by a previous study that rated competencies for evidence-based cancer control [26] and has been used in other assessments of state and local public health practitioners [22, Jacob RR, Allen P, Baker EA, Dodson EA, Duggan K, Fields R, Sequeira S, Brownson RC: **Training needs and supports for evidence-based decision making among the public health workforce in the United States, submitted**]. The 10 EBDM competencies, along with their descriptions as provided on the survey tool, are listed in Table 1.

**Table 1:** **Local health department practitioners’ importance and availability ratings of ten evidence-based decision making (EBDM) competencies**

		**Control (n = 214)**		**Intervention (n = 82)**		**Intervention effect b (SE)** ^**†**^			
		**Pre mean**	**Post mean**	**Pre mean**	**Post mean**	**Unadjusted**		**Adjusted**	
**Prioritization:** *Understand how to prioritize program and policy options.*									
	Importance	8.8	9.2	9.1	9.1	-0.42	(0.19)*	-0.24	(0.21)
	Availability	6.8	7.5	6.4	7.2	0.09	(0.32)	0.22	(0.37)
	Gap	2.0	1.7	2.7	1.9	-0.51	(0.34)	-0.46	(0.40)
**Adapting interventions:** *Understand how to modify programs and policies for different communities and settings.*									
	Importance	8.7	8.8	9.1	9.0	-0.28	(0.22)	-0.21	(0.25)
	Availability	6.3	6.9	5.9	6.6	0.17	(0.31)	0.35	(0.35)
	Gap	2.4	1.9	3.2	2.4	-0.44	(0.34)	-0.56	(0.39)
**Evaluation designs:** *Understand the different designs that are useful in program or policy evaluation.*									
	Importance	8.1	8.4	8.7	8.8	-0.15	(0.22)	-0.17	(0.25)
	Availability	5.5	6.0	5.2	6.3	0.63	(0.34)	0.78	(0.39)*
	Gap	2.6	2.4	3.5	2.5	-0.78	(0.37)*	-0.95	(0.42)*
**Quantifying the issue:** *Understand the uses of descriptive epidemiology (e.g., concepts of person, place, time) in quantifying a public health issue.*									
	Importance	8.4	8.8	8.5	8.8	-0.10	(0.21)	0.03	(0.25)
	Availability	6.8	6.9	6.2	7.0	0.69	(0.35)*	0.78	(0.39)*
	Gap	1.6	1.9	2.3	1.8	-0.80	(0.37)*	-0.78	(0.42)
**Quantitative evaluation:** *Understand the uses of quantitative evaluation approaches (e.g. surveillance, surveys).*									
	Importance	8.4	8.8	8.8	8.9	-0.27	(0.19)	-0.25	(0.22)
	Availability	6.8	7.1	6.8	7.3	0.16	(0.33)	0.48	(0.38)
	Gap	1.6	1.7	2.0	1.6	-0.43	(0.35)	-0.73	(0.40)
**Qualitative evaluation:** *Understand the value of qualitative evaluation approaches (e.g. focus groups, key informant interviews) including the steps involved in conducting qualitative evaluations.*									
	Importance	8.0	8.3	8.5	8.8	-0.03	(0.23)	0.03	(0.26)
	Availability	6.1	6.5	6.2	6.8	0.18	(0.33)	0.32	(0.38)
	Gap	1.9	1.8	2.3	2.0	-0.22	(0.35)	-0.29	(0.40)
**Action planning:** *Understand the importance of developing an action plan for how to achieve goals and objectives.*									
	Importance	8.9	9.1	9.3	9.3	-0.20	(0.17)	-0.06	(0.19)
	Availability	7.2	7.5	7.0	8.0	0.77	(0.31)*	0.98	(0.35)**
	Gap	1.7	1.6	2.3	1.3	-0.97	(0.29)**	-1.04	(0.34)**
**Community assessment:** *Understand how to define the health issue according to the needs and assets of the population/community of interest.*									
	Importance	8.9	9.2	9.4	9.5	-0.21	(0.17)	-0.14	(0.19)
	Availability	7.2	7.6	7.4	7.7	-0.06	(0.29)	0.02	(0.34)
	Gap	1.7	1.6	2.0	1.8	-0.15	(0.30)	-0.16	(0.35)
**Communicating research to policy makers:** *Understand the importance of effectively communicating with policy makers about public health issues.*									
	Importance	8.8	9.0	9.1	9.2	-0.20	(0.20)	-0.19	(0.23)
	Availability	6.2	6.4	5.2	6.3	0.88	(0.35)*	0.86	(0.41)*
	Gap	2.6	2.6	3.9	2.9	-1.08	(0.39)**	-1.05	(0.45)*
**Economic evaluation:** *Understand how to use economic data in the decision making process.*									
	Importance	8.6	8.7	9.0	8.8	-0.32	(0.20)	-0.35	(0.23)
	Availability	5.6	5.6	4.9	5.1	0.24	(0.36)	0.65	(0.41)
	Gap	3.0	3.1	4.1	3.7	-0.56	(0.38)	-1.00	(0.43)*
**Mean of all 10 EBDM competencies**									
	Importance	8.5	8.8	8.9	9.0	-0.22	(0.13)	-0.15	(0.15)
	Availability	6.4	6.8	6.1	6.8	0.37	(0.22)	0.55	(0.25)*
	Gap	2.1	2.0	2.8	2.2	-0.59	(0.23)*	-0.70	(0.27)**

Importance and Availability scores measured on 0-10 scale (greater scores = greater importance/availability); Gap = Importance-Availability.

^†^Unstandardized regression parameter estimate (b) and standard error (SE) for group assignment (Intervention = 1, Control = 0) in simple linear regression model (unadjusted) and multivariate linear regression model (adjusted for job position, population of jurisdiction, highest degree, gender, age, years of public health experience, state); outcome variable is difference score (posttest – pretest); **p-value ≤ 0.01 *p-value ≤0.05.

The entire baseline survey instrument underwent cognitive response testing (n = 12) and test-retest processes (n = 38) for refinement and to document validity and reliability. Cronbach’s alpha values were 0.94 and 0.89 for the importance and availability of EBDM questions, respectively, with 8 of 10 EBDM importance questions having substantial reliability and 7 of 10 availability questions rated with substantial or nearly perfect reliability [27].

Additionally, the intervention group’s post-test questionnaire asked participants to assess how frequently they used EBDM skills and to rate benefits and barriers to using course content. These questions have been used in previous evaluations of the EBPH course [16,19].

### Data collection

All data were collected using Qualtrics survey software [28]. A unique link was emailed to each participant, and non-respondents received email and phone call reminders to bolster response rates. For the control group, baseline data were collected from October 2012 to February 2013 and repeated in October-December 2013. Baseline data were collected from course attendees prior to their trainings and were repeated six months after each training (October – December 2013). Respondents were offered a $20 Amazon gift card incentive for completing the pre-test and a $10 Amazon gift card for completing the post-test. The median pre-test administration time was 14 minutes, and the median post-test was five minutes. Human participant approval was obtained from the Washington University Institutional Review Board.

### Data analysis

An average of 33 participants completed each EBPH course (n_MI_ = 27, n_NC_ = 32, n_OH_ = 33, n_WA_ = 38). Among those invited to complete a post-test (n_control_ = 330, n_intervention_ = 130), data were collected from 236 controls (response rate 72%) and 112 intervention subjects (response rate 86%). Excluding participants who no longer worked at the same organization or who had an undeliverable email address (n_control_ = 22, n_intervention_ = 6), response rates were 77% and 90%, respectively. Efforts were made to update any undeliverable email addresses by contacting the LHDs and by conducting Internet searches for the individual, but survey invitations were not forwarded if the individual was working for a new organization. Although unique survey links should have ensured that the same person completed the pre- and post-test, we compared demographic data from pre- to post-test to determine if survey links were shared without our knowledge. This resulted in the exclusion of 11 cases from the control group. Another 11 controls who did not answer the majority of the EBDM competency questions were also excluded. Among the intervention group, 14 represented state health departments or other non-LHD organizations; they are excluded from all analyses. An additional 16 intervention subjects did not complete a pre-test or did not answer the majority of EBDM competency questions. A total of 214 control and 82 intervention subjects were used in the quasi-experimental analysis (Tables 1 and 2) while the previously mentioned 16 intervention subjects were retained for the analysis represented in Table 3 (n = 98).

**Table 2:** **Characteristics of the sample of local health department practitioners, United States, 2012-2013**

	**Control (n = 214)**		**Intervention (n = 82)**	
	**N**	**%**	**N**	**%**
**Job position**				
Top executive, health officer, commissioner, administrator, deputy, assistant director	93	43.5	16	19.5
Manager of a division or program	79	36.9	27	32.9
Program coordinator, technical expert, other	42	19.6	39	47.6
**Population of Jurisdiction**				
<25,000	24	11.2	6	7.3
25,000 – 49,999	52	24.3	12	14.6
50,000 – 99,999	43	20.1	18	22.0
100,000 – 499,999	75	35.0	37	45.1
500,000+	20	9.3	9	11.0
**Highest degree**				
Doctoral	16	7.5	0	0
Master of Public Health	40	18.7	24	29.3
Other masters degree	57	26.6	29	35.4
Nursing	42	19.6	4	4.9
Bachelors degree or less	59	27.6	25	30.5
**Gender**				
Male	73	34.1	9	11.0
Female	141	65.9	73	89.0
**Age**				
20 – 29	9	4.2	10	12.2
30 – 39	27	12.6	30	36.6
40 – 49	52	24.3	15	18.3
50 – 59	80	37.4	26	31.7
60+	46	21.5	1	1.2
**Years in public health**				
Mean (St. Dev)	17.9	(9.90)	12.4	(7.87)

**Table 3:** **Local health department respondents’ use of Evidence-Based Public Health (EBPH) course content (n = 98)**

	**N**	**%**
**On average, every month since the EBPH course I have:**		
Searched the scientific literature for information on programs.	35	35.7
Used the EBPH materials/skills in planning a new program.	26	26.5
Used the EBPH materials/skills in modifying an existing program.	24	24.5
Used the EBPH materials/skills in evaluating a program.	23	23.5
Referred to the EBPH readings that were provided.	22	22.4
Used the EBPH materials/skills for grant applications.	3	3.1
**The EBPH course content helped me:**		
See applications for this knowledge in my work.	91	92.9
Become a better leader who promotes evidence-based decision making.	85	86.7
Acquire knowledge about a new subject.	84	85.7
Make scientifically informed decisions at work.	79	80.6
Communicate better with co-workers.	64	65.3
Read reports and articles.	62	63.3
Adapt an intervention to a community's needs while keeping it evidence based.	62	63.3
Develop a rationale for a policy change.	61	62.2
Teach others how to use/apply the information in the EBPH course.	60	61.2
Identify and compare the costs and benefits of a program or policy.	59	60.2
Implement evidence-based practices in CDC cooperative agreement or other funded programs.	50	51.0
Obtain funding for programs at work.	39	39.8
**I have not used the EBPH course content as much as I would like because:**		
The people I work with do not have EBPH training.	48	49.0
There is not enough funding for continued training in EBPH.	40	40.8
I do not have enough time to implement EBPH approaches.	40	40.8
There was too much information and not enough time to process it.	23	23.5
Within my agency there are no incentives to use EBPH.	21	21.4
I still lack sufficient skills in EBPH.	17	17.3
My organization does not have a culture that supports the use of EBPH approaches.	11	11.2
The information lacked relevance.	5	5.1
The information was too complex.	4	4.1

Respondents rated perceived importance followed by availability of each EBDM competency. Availability was defined as ‘how available you feel each skill is to you when you need it (either in your own skillset or among others’ in your agency).’ Importance and availability were measured on a continuous 11-point scale in which only the endpoints were defined (0 = unimportant/not available, 10 = very important/available). A ‘gap’ score was computed by subtracting each availability score from its corresponding importance score. A net difference was calculated for importance, availability, and gap scores by subtracting the pre-test score from the post-test score for each respondent. Difference scores were normally distributed and were used as the outcome variable in simple linear regression models. The estimated regression coefficient of a group assignment variable (coded as intervention = 1, control = 0) represented the average change in the outcome variable associated with the intervention. Standard multiple linear regression models adjusted for job position, population of jurisdiction, highest degree, gender, age, state, and years of public health experience. Frequency of EBPH skill use was measured as weekly, monthly, quarterly, and seldom/never. Benefits and barriers were measured on a 5-point Likert scale, and combined ‘agree’ and ‘strongly agree’ categories are reported. Chi-square tests assessed differences between categorical groups.

## Results

Table 2 shows post-test demographic characteristics of control and intervention respondents used in the quasi-experimental analysis. In general, controls had higher-level jobs, were more likely to be older and male, and had more years of public health experience than intervention subjects (p < 0.01). Population of jurisdiction was roughly balanced between the two groups (p = 0.26). Over one-half of both groups had attained post-graduate degrees (53% of control, 65% of intervention). Control and intervention respondents did not significantly differ from non-respondents (p < 0.05) for any of the variables listed in Table 2.

Controls (n = 214) represented 32 U.S. states, averaging 6.9 respondents per state (St. dev. 4.9) and including respondents from the four intervention states who were unassociated with the training (n_MI_ = 4, n_NC_ = 7, n_OH_ = 10, n_WA_ = 7). All 27 states in which all LHDs are locally governed were represented, and locally governed LHDs from 5 of 13 mixed governance states were represented. Intervention states were represented approximately equally (n_total_ = 82, n_MI_ = 22, n_NC_ = 21, n_OH_ = 22, n_WA_ = 17) in the quasi-experimental analysis.

All pre-test mean importance scores for the 10 EBDM competencies were 8.0 or greater on the scale of 0 – 10 for both groups, leaving little room for improvement (moreso in the intervention group with higher pre-test means than the control group in all 10 competencies) (Table 1). While nearly all mean importance scores improved from pre-test to post-test in both groups, negative mean difference scores indicate the greater increase in control scores relative to intervention scores. No adjusted scores, and only one unadjusted score (‘prioritization’), showed significant differences between groups.

Availability of EBDM competencies increased more for the intervention group, relative to the control group, for unadjusted and adjusted measures of all 10 competencies (except the unadjusted measure of ‘community assessment’). The overall post-test availability means of all 10 competencies were equivalent, with the intervention group starting lower at pre-test. Adjusted mean differences were significant (p < 0.05) for ‘action planning,’ ‘communicating research to policy makers’, ‘evaluation design’, ‘quantifying the issue’, and the overall mean availability score. Smallest availability increases between groups were in ‘community assessment’ and ‘prioritization’.

Gaps between the importance and availability of each EBDM competency decreased in all 10 competencies and in the overall mean, with significant (p < 0.05) decreases found in: ‘evaluation design’, ‘action planning’, ‘communicating research to policy makers’, ‘economic evaluation’, and the overall mean. The adjusted estimates of ‘quantifying the issue’ and ‘quantitative evaluation’ approached significance (p = 0.07). The smallest gap decreases between groups were in ‘community assessment’ and ‘qualitative evaluation’.

Over 60% of EBPH course attendees reported using EBPH materials and skills at least quarterly in planning, modifying and evaluating programs, in searching scientific literature, and in referring to course readings. Between 22% and 36% of EBPH course attendees reported using course materials or skills on at least a monthly basis in these same five categories (Table 3). In three categories (planning, modifying, and evaluating programs), participants without post-graduate degrees were more likely to report monthly use (p < 0.05). The majority of participants indicated agreement with 11 of the 12 benefits statements (excluding only obtaining funding, 39.8%). Highest rated benefits were: acquiring new knowledge and seeing applications for it in their work, becoming better leaders, and making scientifically informed decisions. The largest barriers to using course content included lack of time for implementation, lack of funding to continue training, and co-workers not being similarly trained. Importantly, only 17.3% of participants did not use course content because they lacked sufficient skill to do so.

Nearly 45% of participants indicated that EBDM had increased within their agency since completing the EBPH training. An open-ended survey question solicited examples, and common themes included: selecting new programs based on scientific literature, epidemiologic data, and tools such as *The Guide to Community Preventive Services*; critically evaluating current programs and modifying or eliminating programs as necessary; writing grants to secure new funding; conducting evaluation, community health assessments, and strategic planning; supporting health department accreditation processes; and providing a framework for talking with leaders. One participant noted:

- ‘It helped raise awareness about evidence based decision-making among agency leadership, paving the way for those of us who completed the training to discuss, promote and facilitate integration of it in our public health programming, services, grant writing etc. and receive increased support to do so. It assisted in it becoming part of a common organizational language.’

## Discussion

This study shows the potential for improving LHD practitioners’ capacity in EBDM using a train-the-trainer approach involving diverse partners. The EBPH course, developed by the PRC-StL, has been previously evaluated [16,19,20], but this quasi-experimental design (pre/post with external comparison group) improves the quality of the evidence [29], examining the potential effects of the training while accounting for secular trends and other external factors.

Partners within four states tailored and delivered a previously established EBPH curriculum and provided technical assistance to course participants. Both control and intervention groups saw mean increases in importance and availability scores (and decreases in gap scores), possibly indicating the increased focus on EBDM from other sources, such as funding and accreditation agencies. However, the intervention group consistently saw greater gains in availability of EBDM competencies and decreased gaps between importance and availability, particularly in the areas of ‘action planning’, ‘communicating research to policy makers’, ‘evaluation design’, ‘quantifying issues (using descriptive epidemiology)’, and ‘economic evaluation’. Importance of EBDM competencies showed little change between pre- and post-assessments, likely due to their already high ratings at baseline and indicating agreement with the procedure by which these competencies were developed (*i.e.*, competencies were originally selected and prioritized as those that were important) [26].

Across four surveys of state and local health department practitioners (including the baseline surveys from which control subjects of this current study were selected) and consistent with previous research [22], the largest gaps between the importance and availability of EBDM competencies were consistent across samples: ‘economic evaluation,’ ‘communicating research to policy makers,’ ‘evaluation designs,’ and ‘adapting interventions’ [Jacob RR, Allen P, Baker EA, Dodson EA, Duggan K, Fields R, Sequeira S, Brownson RC: **Training needs and supports for evidence-based decision making among the public health workforce in the United States, submitted**]. The current evaluation showed significant decreases in gaps in the first three, indicating that the EBPH course is targeting the areas of EBDM most in need of improvement. Participants in the EBPH course in this multi-state intervention also showed similar use of skills and agreement with benefits and barriers to using course material as did almost 500 previous participants of the course who were primarily taught by faculty associated with the course’s original developers [16,19].

### Lessons learned

Based on this evaluation, EBPH training courses can effectively improve the availability of several skills essential to EBDM among LHD practitioners. With the development of successful partnerships and the availability of experienced trainers, such a course can be tailored and replicated in nearly any environment. Based on the experiences of the trainers and on participants’ onsite evaluations of the course, we share below some lessons learned from the adaptation and implementation of the EBPH course in this train-the-trainer model:

Some participants found components of the curriculum to be too elementary while others with less experience or formal training learned new skills. Efforts should be made to assess the audience’s level of knowledge during the planning phases of the course and adapt course content to the appropriate level of knowledge and expertise. However, a heterogeneous group supports networking among individuals in different roles (*e.g.*, evaluators, surveillance staff, health educators), and this heterogeneity also reflects the realities of staff expertise within departments and programs. Not every practitioner must possess every EBDM skill. Rather, as a whole, the team should be able to conduct an EBDM process. More experienced participants could be asked to self-identify and support less experienced participants during vital program exercises.

Two states incorporated web-based technology to deliver three course modules, and the majority of their participants found the webinars to be useful and to enhance learning. Webinar formats can increase reach and sustainability, and participants appreciated the flexibility they afford. However, strengths of in-person training as identified by the participants (*e.g.*, interacting with new peers, working through examples face-to-face, hearing about best practices from other counties) are difficult to recreate in web-based formats.

Similar to previous evaluations of the course [16,20], participants requested more specific examples of how to apply an evidence-based process to practical work, more tailored materials (to their specific program areas), and more problem sharing amongst course participants. They appreciated hands-on activities and exposure to new resources and take-home tools. If possible, it is recommended to have previous course attendees share experiences in using the new knowledge and making changes within their agency.

Participants consistently requested more guidance on economic evaluation. This competency also had the lowest mean pre- and post-test availability scores among both control and intervention groups. Participants may benefit from a more simplified approach to presenting this content, with a greater focus on accessing, rather than conducting, economic evaluations.

Curriculum related to the competencies with low availability gains and small decreases in gap scores (*e.g.*, ‘community assessment,’ ‘qualitative evaluation’) should be reviewed for opportunities to incorporate new tools, exercises, or teaching points. In some cases, low availability gains may reflect existing training efforts in that area (*e.g.*, a state health department has invested in community assessment trainings) and the EBPH curriculum should be coordinated with those existing efforts.

Trainings were strengthened by the participation of trainers with a diversity of experience and expertise and by coordination among presenters in advance of the training to ensure consistent messaging and localization of data and concepts.

Having teams of two or three individuals from an agency attend the course together creates a ‘critical mass’ of trained staff in an agency [30,31] and enhances the likelihood of influencing the agency’s decision-making processes.

A focus on training leaders with targeted or more advanced EBDM sessions is also important. Leadership buy-in is critical when building skills, fostering expectations for EBDM, and conducting participatory decision-making [32-34].

### Next steps

With promising results from the implementation of this intervention, a next step is to identify practices for further scaling up EBDM capacity-building efforts among the nation’s 2,565 LHDs. Health departments, particularly those applying for PHAB accreditation, need to enhance their workforce’s capacity to implement EBDM. The effectiveness of webinar formats should be investigated, as they can be an efficient way of addressing the increasing demands placed on public health professionals as they face declining government funding, staff reductions, and travel restrictions.

Our study was not designed to test webinar effectiveness. Only two EBDM competencies could be related to EBPH modules delivered via webinar (Module 3: Quantifying the Issue for Michigan and Module 7: Economic Evaluation for Michigan and Washington). An assessment of the related competency differences in importance, availability and gaps for participants from these states versus others yielded no significant findings (p < 0.05). While we cannot draw conclusions due to small sample sizes, these findings may imply that webinars were as effective as in-person training. It is currently unknown if web-based public health training is as effective as in-person training, and further research is indicated.

Effective webinar development can incorporate adult learning principles, focusing on scenario-based (rather than lecture-based) learning and thereby increasing participants’ engagement and ability to apply lessons to their work [35]. Maintaining the local tailoring of course material for webinar development may sustain some of the advantages (*e.g.*, locally relevant examples and credible, familiar trainers) experienced in this trial.

### Limitations

Some limitations of this study should be noted. Ideally, control and intervention groups would have been retested within the same time intervals; the timeframe of this research project did not allow for that. Training course participants may have been more biased towards socially desirable responses than control subjects. Intervention and control groups could have differed on more demographic variables than those measured and accounted for in adjusted models. This study was restricted to localized, or decentralized, governance structures, and results do not necessarily apply to other types of LHDs (*i.e.*, those that are part of state government).

## Conclusions

This evaluation shows the value and effectiveness of an EBDM capacity building course among local public health practitioners using a train-the-trainer approach and thus extending the reach of the course. The PRC-PHTC/PBRN partnership network covers LHDs in 28 states, expanding the potential reach of a scaled-up version of this project. This approach allows for local tailoring of strategies, examples and exercises, and it provides familiar and credible trainers who remain available to participants for technical assistance.
